# Diversity and population structure of red rice germplasm in Bangladesh

**DOI:** 10.1371/journal.pone.0196096

**Published:** 2018-05-02

**Authors:** M. Z. Islam, M. Khalequzzaman, M. F. R. K. Prince, M. A. Siddique, E. S. M. H. Rashid, M. S. U. Ahmed, B. R. Pittendrigh, M. P. Ali

**Affiliations:** 1 Genetic Resources and Seed Division, Bangladesh Rice Research Institute, Gazipur, Bangladesh; 2 Department of Entomology, Michigan State University, East Lansing, MI, United States of America; 3 Entomology Division, Bangladesh Rice Research Institute, Gazipur, Bangladesh; National Cheng Kung University, TAIWAN

## Abstract

While the functionality and healthy food value of red rice have increased its popularity, such that market demand for it is expected to rise, most strains suffer from low grain yield. To perform diversity and population structure analyses of red rice germplasm, therefore, becomes essential for improving yields for commercial production. In this study, fifty red rice germplasm from the Bangladesh Rice Research Institute (BRRI) genebank were characterized both morphologically and genetically using fifty simple sequence repeat (SSR) markers. Overall, 162 alleles were detected by the markers with the detected allele number varying from two to seven. Additionally, 22 unique alleles were identified for use as a germplasm diagnostic tool. The highest and lowest polymorphic information content (PIC) indices were 0.75 and 0.04 found in markers RM282 and RM304, respectively, and genetic diversity was moderate, varying from 0.05 to 0.78 (average: 0.35). While phylogenetic cluster analysis of the fifteen distance-based agro-morphological traits divided the germplasm into five clusters (I, II, III, IV and V), a similar SSR analysis yielded only three major groups (I, II, and III), and a model-based population structure analysis yielded four (A, B, C and D). Both principal component and neighbors joining tree analysis from the population structure method showed the tested germplasm as highly diverse in structure. Moreover, an analysis of molecular variance (AMOVA), as well as a pairwise FST analysis, both indicated significant differentiation (ranging from 0.108 to 0.207) among all pairs of populations, suggesting that all four population structure groups differed significantly. Populations A and D were the most differentiated from each other by FST. Findings from this study suggest that the diverse germplasm and polymorphic trait-linked SSR markers of red rice are suitable for the detection of economically desirable trait loci/genes for use in future molecular breeding programs.

## Introduction

Rice is the main food for more than half of the world’s population and is the staple food for the people of Bangladesh, constituting over 91% of the food grain production, and providing 62% of the caloric as well 46% of the protein intake in the average daily diet [1]. The rice production area in Bangladesh is approximately 11.4 million hectares (ha) producing 51.64 million tons of rice annually [2].

Bangladesh has three major rice crops, Aman, Aus, and Boro, which are grown during three overlapping seasons and with a large number of varieties suitable for various agro-ecological and climatic niches [3]. The Aus growing season usually starts in March-April, is harvested in July-August, and has an average yield of 2.00 t/ha, which is lower than the other two crops. More than 30% of Aus rice in Bangladesh are a red variety, which characteristically has limited yield. In Bangladesh, high temperatures during the Aus growing season increase plant height but decrease growth duration in rice. Solar radiation is also lower in Aus versus Boro growing seasons, resulting in lower Aus yields. Traditional red rice varieties also typically have weak stems, low tillering ability, long droopy leaves, turn yellow during grain development, and become logged at maturity. However, farmers still plant them widely because they can be grown under low inputs and produce a reasonable yield under the seasonal environmental conditions to which modern varieties are not adapted. Nonetheless, this limited yield makes farmers less interested in cultivating red rice despite its high medicinal value and use as a functional food.

Modern breeding techniques could improve the yield of red rice to overcome this problem. Successful breeding for crop improvement, however, depends on genetic variability in the parents [4], such that a lack of genetic variability would have the potential to significantly limit breeding progress and/or yield and quality crop improvements [5]. Increased knowledge into the genetic diversity of any germplasm collection, therefore, enhances the possibility of crop improvement and the development of superior cultivars.

Very few studies, however, have investigated the genetic diversity of Aus rice in Bangladesh [6–8]. While more comprehensive research into genetic variability, as well as the population configuration, of Aus varieties would inform germplasm utilization for rice breeding, research into morphological and agronomic variation will also be needed for any future breeding program. Simple sequence repeats (SSRs)—a form of both allele-specific and co-dominant molecular marker—has shown considerable potential for determining genetic variability and any significant levels of allelic polymorphism [9, 10]. Moreover, to identify diverse crop genotypes using phenotypic as well as genotypic markers is more beneficial than conventional approaches [11]. For instance, SSRs have been used for genetic variability assessment, molecular characterization, genotypic identification, and population structure estimations in multiple previous investigations of rice genetics [12–18]. SSR markers have also proven useful for diversity studies [10, 19], construction of linkage maps [20, 21], and for QTL analysis [21, 22] in rice.

Clustering a large number of germplasms represents a simple and effective process for assessing the genetic variability of germplasm [23], along with other statistical approaches such as population structures [24], principal component analysis (PCA) [25], principal coordinate analysis (PCoA) [26], and AMOVA. Structure analysis of natural populations by molecular markers is also commonly utilized globally for diverse crops, such as rice [27, 28], apple [29], peanut [30], sweet sorghum [31], wheat [32], and cucumber [32].

Cultivars developed using natural populations and germplasm can have unique target characteristics for attaining high yields and stress tolerance. Analyzing genetic structures, as well as identifying allelic variation among and/or within a population, represents an important approach for the design of a molecular breeding program [33]. Analyzing population structures, then, is a prerequisite for association analysis and is considered a key for avoiding red herrings while constraining the association analysis of any germplasm [23, 24, 34].

This study uses red rice to perform a diversity analysis using both morphological and molecular approaches. In general, red (and black) rice are staple foods that an increasing number of consumers are demanding due to their documented health-promoting benefits [35]. These include the provision of anthocyanin, which is beneficial not only as an antioxidant [36–39] and anti-inflammatory [40] but has also been linked to anti-carcinogenic properties [41, 42]. These rices may also be helpful for the prevention or cure of diseases caused by vitamin A and B deficiencies [43]. Besides its high functional food value, the pericarp of red rice has a high measured content for total soluble phenolic compounds (81%) and antioxidant properties [44]. Comparative soluble phenolic compound levels of only 38–60% have been observed in light brown rice grains [44, 45].

Commercial availability of red rice in markets is limited, however, by a lack of improved varieties at the producer level. Few farmers cultivate this rice using local varieties, due to low yields, long durations, and susceptibility to lodging [18]. Moreover, although some of the local red upland rice germplasm have been collected from different regions in Bangladesh and conserved in the BRRI genebank, studies to date on the genetic diversity of this invaluable upland red rice collection using molecular markers in Bangladesh have been inadequate. Given that this collection provides an invaluable genetic resource of pre-breeding materials useable for varietal improvement [46], the present study set out to characterize the genetic variability, as well as the population structure, using fifty polymorphic SSR markers on fifty red rice germplasm grown at upland conditions in Bangladesh.

## Materials and methods

### Plant materials

**Fig 1:**
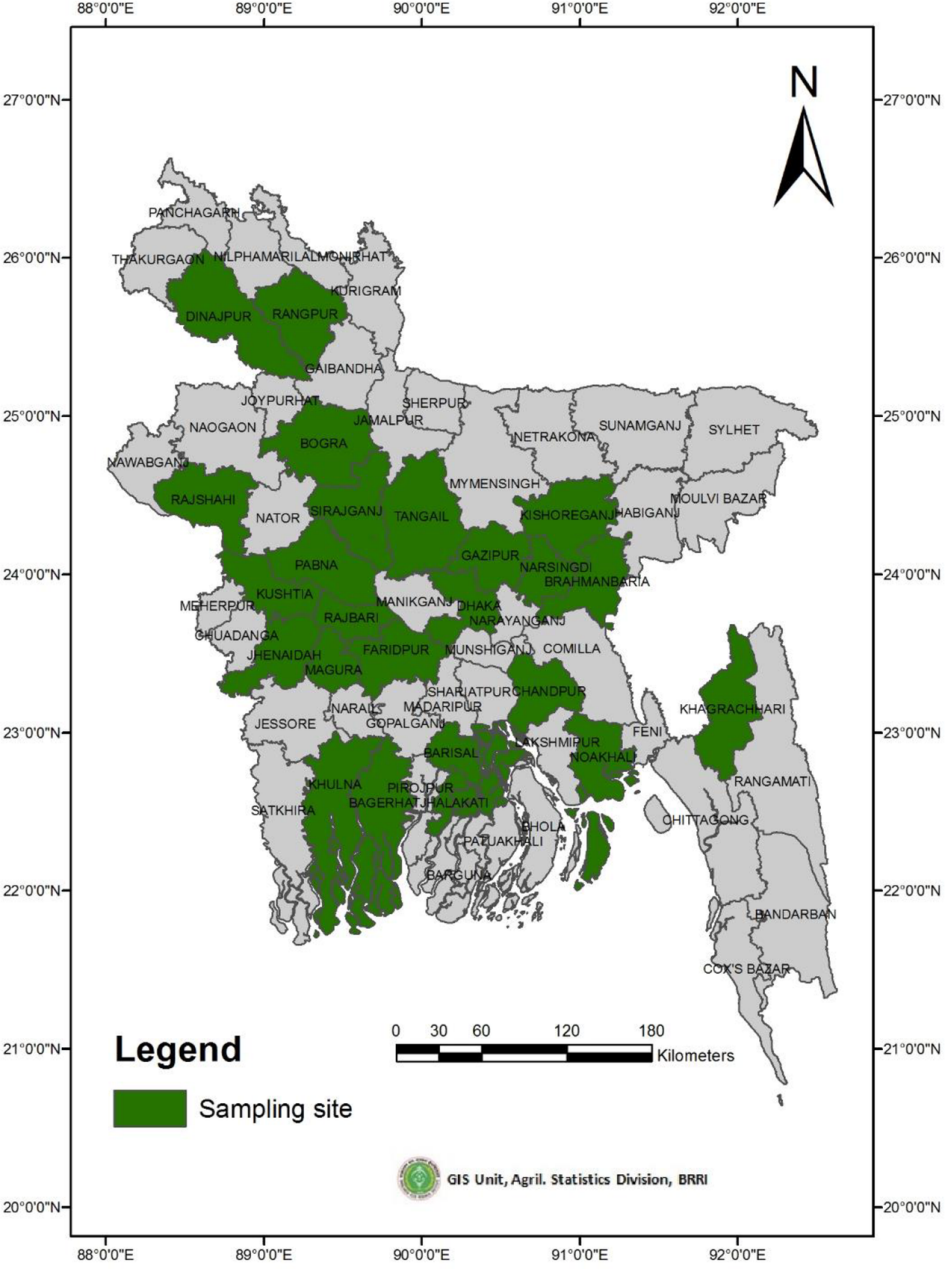
Map of geographic location of germplasm collection.

**Fig 2:**
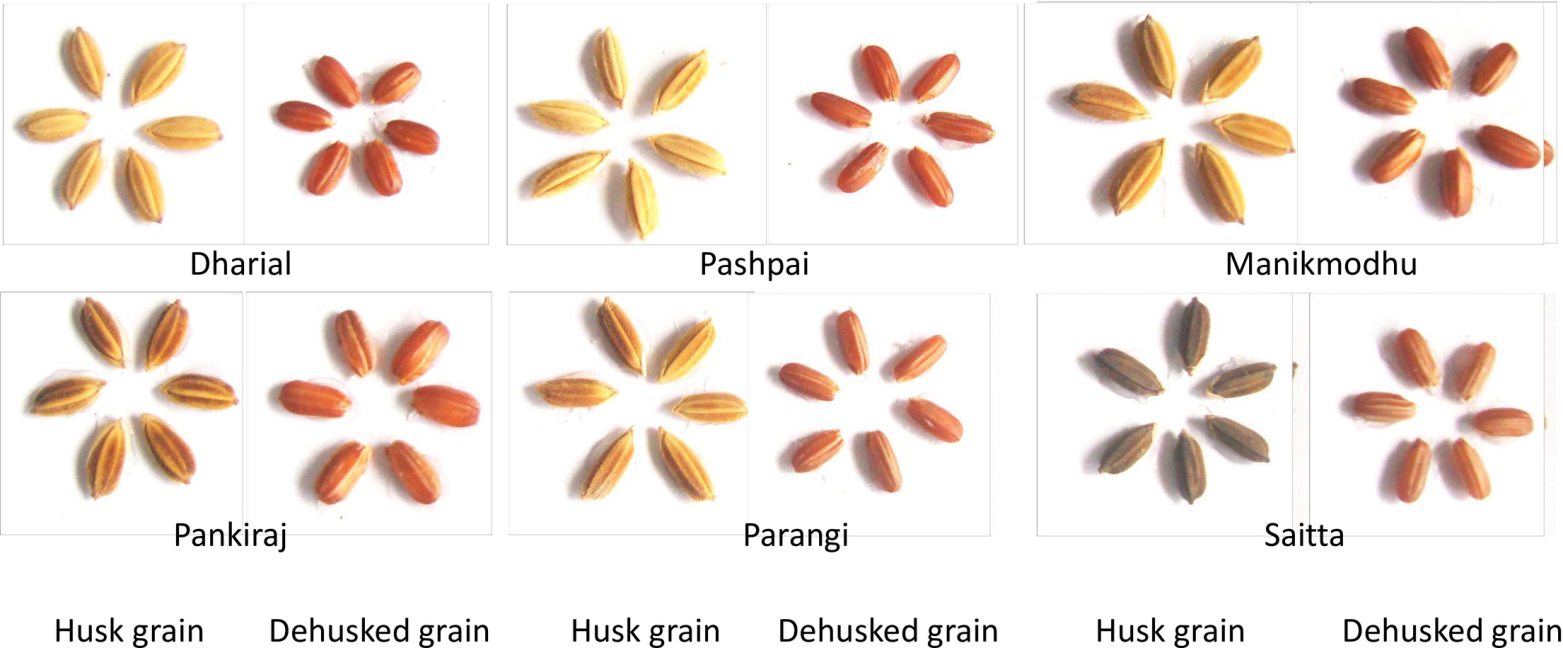
Selection of tested red rice among the tested germplasm showing colored pericarp pattern.

Rice germplasm, including Aus, Aman and Boro rice cultivated throughout Bangladesh, have been collected and conserved by the Bangladesh Rice Research Institute (BRRI) genebank. To date, approximately 8,200 germplasm have been preserved by the BRRI genebank, of which 1,500 are Aus rice, with more than 30% of those considered red rice. For this study, we cultivated fifty red rice germplasm—collected by BRRI from the sites noted in Fig 1 and designated as red due to their red-colored pericarp (see Fig 2)—in upland conditions during the Aus growing season in Bangladesh.

### Agro-morphological characterization

All experiments described here were conducted at the Bangladesh Rice Research Institute in Gazipur, Bangladesh during the March to June Aus rice growing season in 2015. Fifty red rice germplasm were seeded directly under upland conditions in two 5 m x 3 m plot replicated trials. Production practices, including fertilizers, intercultural operations, pest control, and grain harvesting, were conducted according to standard methods.

Fifteen agro-morphological traits—leaf area index, culm diameter, effective tiller number, days to flowering, plant height, days to maturity, five panicles weight, filled grains per panicle, unfilled grains per panicle, grain length, grain breadth, 1000 grain weight, length-breadth ratio, and yield per hill—were recorded. Genetic diversity was worked out for the principal component analysis [47] and Mahalanobis’ generalized distance (D^2^) analysis [48]. All multivariate analyses were performed using the GENSTAT 5.5 program.

### Molecular characterization

We used fifty well-distributed SSRs for the diversity analysis; position (cM), repeat motifs, and chromosomal positions for the SSR markers can be found in the rice genome database [49]. Most of these markers were obtained from a panel of fifty standard SSR markers, which has been proposed by CGIAR for rice diversity analysis [50]. DNA was extracted from the young leaves of 14-day-old plants using the miniscale method [51]. The total PCR reaction was performed using the standard procedure. The 10 μL of each PCR product, with 2μL of a loading dye, were analyzed using 8% polyacrylamide gel electrophoresis in a 1 × TBE buffer, run at 75 V for between 2.0–2.5 h depending upon the allele size. The gels were then stained, using ethidium bromide solution (0.5 mg/mL), for 25 min and exposed to UV light using a molecular imager gel documentation unit (XR System, Uvitec Cambridge, France) for visualization.

### Data analysis

The band-size for each of the markers was scored using the AlphaEaseFC 4.0 software. Using PowerMarker version 3.25 [52], summary statistics included the following: the number of alleles, the major allele size and its frequency, gene diversity, and the polymorphism information content (PIC) value. For the unrooted phylogenic tree, the genetic distance was calculated using MEGA 6 based on Nei’s unbiased pairwise [53, 54]. Binary form for allele frequency was prepared using PowerMarker software and used for dendrogram construction by NTSYS-pc software [25]. The unweighted pair grouping method using arithmetic average (UPGMA) was used to determine a similarity matrix following the Dice coefficient with the SAHN subprogram.

Population STRUCTURE for germplasm was determined using STRUCTURE, (version 2.3.4) [24, 55]. The number of clusters (K) investigated, in this study, ranged from one to fifteen, with five replications for analysis of each K value. The model following admixture and correlated allele frequency with a 5,000 burn period and a run length of 50,000 were used for conducting model-based structure analysis. Output of analysis was collected using the STRUCTURE harvester [56] and identified 4 as the best K value based on the LnP(D) and Evanno’s ΔK [57]. Principal components analysis (PCA) analysis was conducted also using the STRUCTURE software. In order to summarize the major patterns of variation within the multi-locus dataset, an analysis of molecular variance (AMOVA) using GenAlEx V6.5 [26] was also performed.

## Results

### Morphological diversity

**Fig 3:**
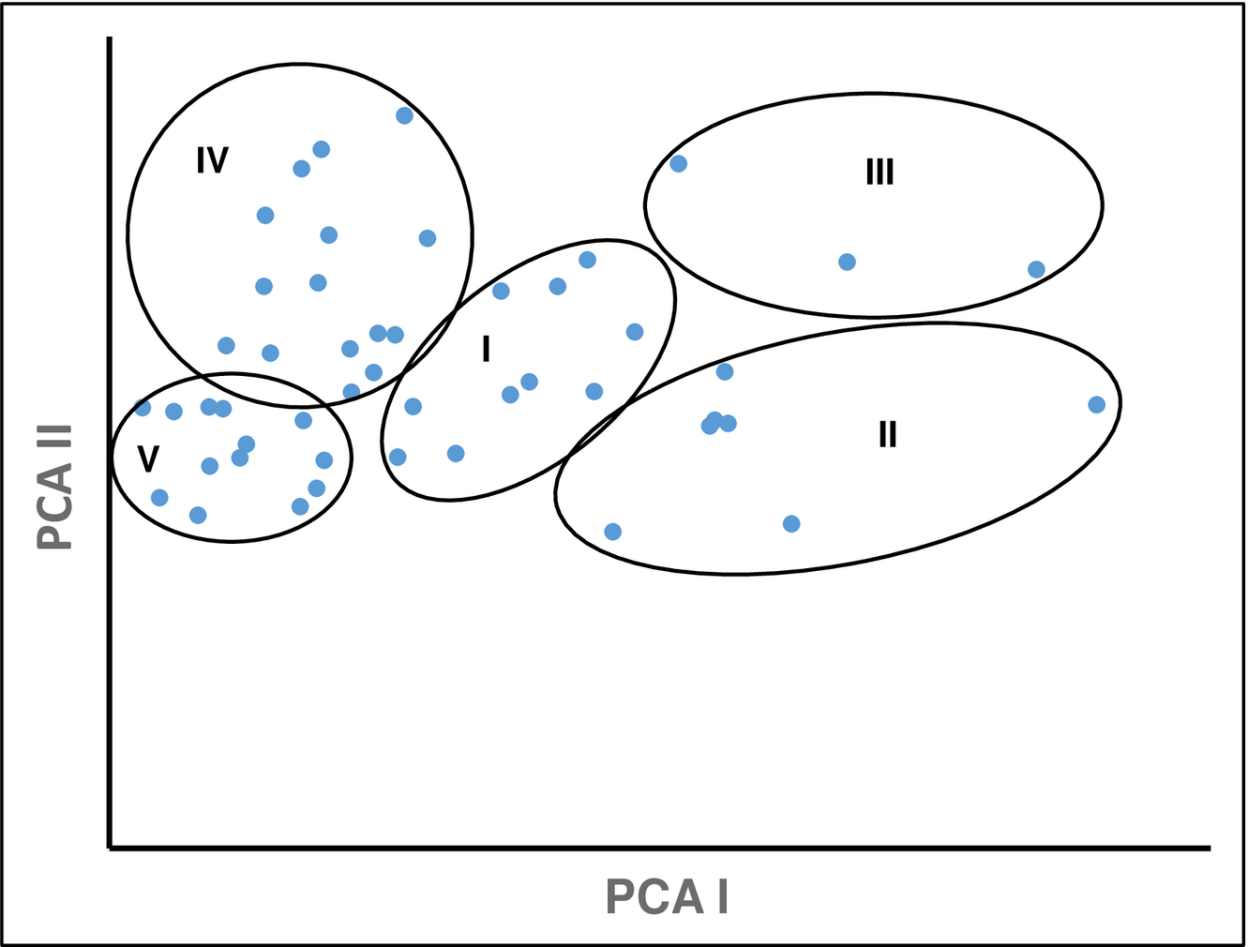
Principal component analysis (PCA) of fifty red rice germplasm based on morphological traits.

Analysis of variance (ANOVA) demonstrated significant variation among fifty red rice germplasm for all fifteen agro-morphological traits. Using PCA, we observed that the first axis mainly accounted for the variation in the germplasm (27.89%) followed by second axis (18.41%). Our results also showed that the first six components with vector values >1 accounted for 80.08% of total variation, with components PCA3, PCA4, and PCA5 contributing 11.26%, 9.77%, and 6.08%, respectively. Using the first two components, Fig 3 shows the distribution pattern of five groups. Based on the non-hierarchal clustering method, using Mahalanobis’ D^2^ statistic, the fifty red rice germplasm with fifteen agro-morphological traits grouped into five clusters (I, II, III, IV, V) (see Table 1). The clustering pattern also revealed that germplasms constellated into the same clusters did not originate from similar geographic regions. The maximum number of germplasm (16) were grouped in cluster IV, followed by thirteen in cluster V, ten in cluster I, eight in cluster II, and the minimum (3) in cluster III.

**Table 1:** List of red rice germplasm including geographic location and clustering group based on morphology and model based STRUCTURE analysis.

SL No.	Genotypes	Acc. No.	Location	Group Based by STRUCTURE Analysis	Cluster Group by Morphology
1	Dharial	18	Dhaka	I	V
2	Dular	22	Dhaka	I	V
3	Hashikalmi	30	Dhaka	III	IV
4	Panbira	50	Dhaka	I	II
5	Pashpai	51	Dhaka	II	IV
6	Pukhi	55	Dhaka	III	V
7	Surjamukhi	60	Dhaka	I	II
8	Laksmilata	67	Dhaka	III	IV
9	Kalamanik	68	Dhaka	III	V
10	Burikatari	150	Tangail	II	IV
11	Manikmodhu	572	Rangpur	III	I
12	Kadamoni	573	Rangpur	III	V
13	Honumanjata	574	Rangpur	IV	V
14	Begunbahar	651	Brahmanbaria	II	V
15	Noyantara	654	Brahmanbaria	I	II
16	Matichak	1199	Faridpur	III	IV
17	Benaful	1529	Dinajpur	III	III
18	Binnatoha	1622	Noakhali	II	IV
19	Pankiraj	1700	Faridpur	III	V
20	Baismuguria	1701	Faridpur	III	V
21	Benamuri	1732	Khulna	I	I
22	Chapla	1961	Kishoreganj	III	IV
23	Pakiraj	1967	Kishoreganj	III	I
24	Kalisaita	2102	Dhaka	III	V
25	Surjamukhi	2114	Narsingdi	I	II
26	Madabjata	2118	Gazipur	III	V
27	Begunbichi	2120	Dhaka	I	V
28	Jameri	2182	Bogra	I	I
29	Botwessor	3235	Dhaka	I	I
30	Sarisaful	3239	Gazipur	I	III
31	Khorajumri	3519	Pabna	III	I
32	Beursona	3523	Sirajganj	III	IV
33	Saita	3543	Jhalakati	III	IV
34	Dhalisaita	3551	Barisal	II	IV
35	Sreerampuri	3560	Barisal	III	IV
36	Narikeljhupi	3721	Rajshahi	III	II
37	Binnasopa	3734	Barisal	II	I
38	Manikjor	4028	Jhenaidaha	III	V
39	Boilam	4608	Noakhali	II	II
40	Tusha	4623	Kustia	III	IV
41	Parangi	4644	Magura	III	IV
42	Beerpala	4954	Khulna	I	IV
43	Kharojamri	4996	Pabna	IV	I
44	Kerondol	6210	Bhola	II	I
45	Khatomala	6622	Bagerhat	IV	I
46	Fullbadam	7266	Khagrachhari	I	II
47	Moisor	7420	Barisal	II	I
48	Laithajhora	7817	Chandpur	IV	II
49	Rateil	7852	Barisal	II	IV
50	Shibjhota	7854	Rajbari	I	III

**Fig 4:**
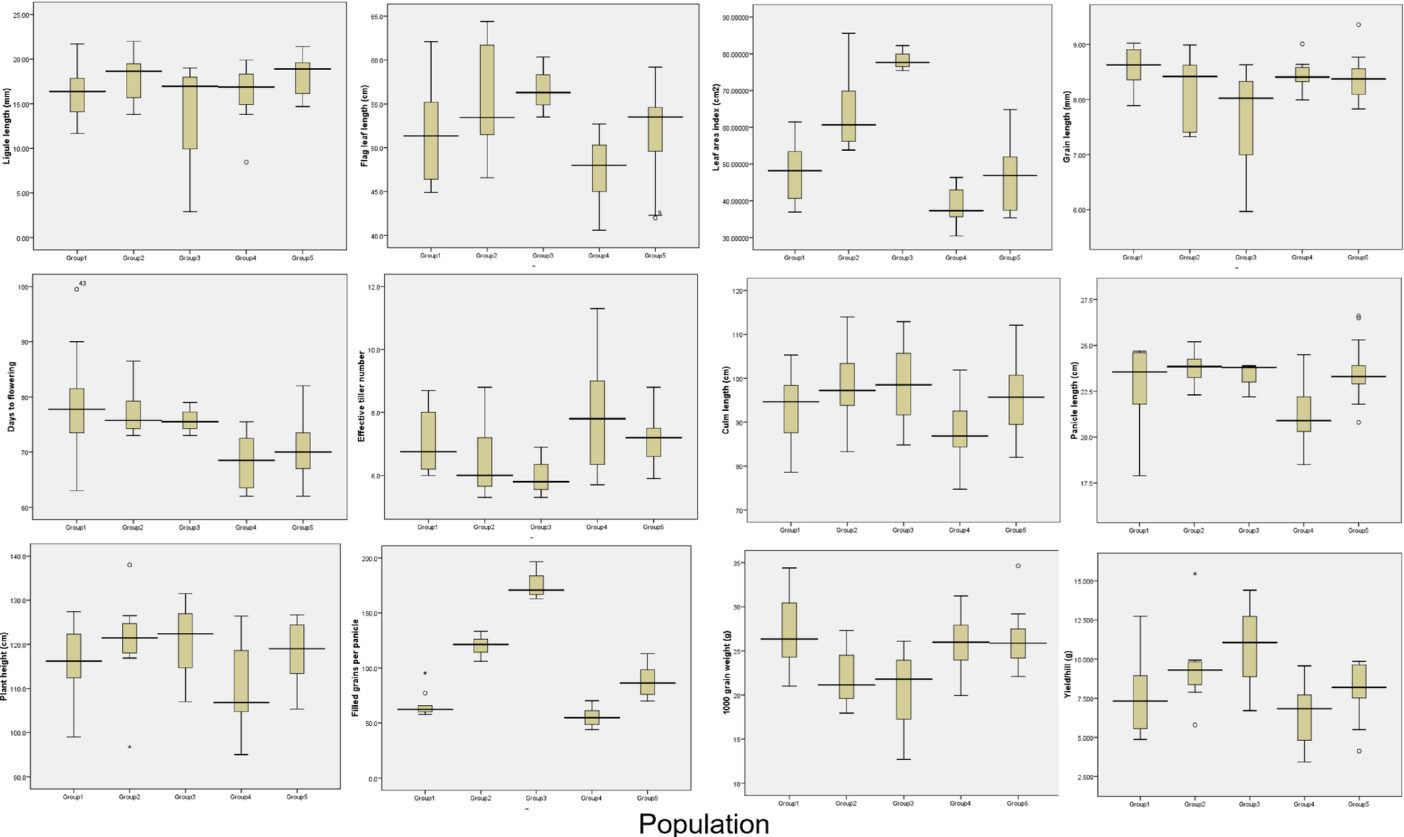
Distribution of twelve quantitative morphological traits in five group populations derived from cluster analysis.

Fig 4 displays the frequency distribution of twelve quantitative morphological traits among the five groups of populations obtained from cluster analysis. The Group3 population showed the highest plant height, while the Group5 population showed the lowest plant height. The greatest variation was observed in filled grain per panicle among the groups, with the Group3 population showing the highest number of filled grain per panicle. Yield is the most important trait for breeding purposes. The Group3 population showed the highest yield per hill while Group4 showed the lowest (Fig 4). These results revealed that significant variation existed between the five groups of red rice germplasm generated from different ecological origins in Bangladesh. Overall, the yield per hill, panicle length, days to maturity, plant height, and leaf area index were 3.40–15.45 g, 17.90–26.30 cm, 90–128 days, 95–138 cm, and 30.43–85.56 cm^2^, respectively.

Average intra- and inter-cluster distance for the entire germplasm based on phenotypic traits was determined. Considerable variation occurred in intra-cluster distances (ranging from 0.65 in cluster II to 1.24 in cluster III), indicating wider diversity prevailing among germplasm within clusters. The germplasm under cluster III (1.24) was the most heterogeneous; germplasm under cluster II (0.65) was comparatively homogenous or less diverse. Inter-cluster distances ranged from 3.70 to 16.31, ranging from a maximum in clusters IV and III (16.31), clusters III and I (14.98), and clusters V and III (12.23), indicating a wider genetic diversity among the germplasm between these groups. In contrast, we observed the lowest inter-cluster distance between clusters I and IV (3.70) and clusters I and V (4.49), indicating a narrower, genetically close, diversity. In general, the germplasm comprising clusters IV and III were more diverse than those of clusters I and IV. Overall, cluster IV had the highest inter-cluster distances compared to other clusters, indicating wider genetic germplasm diversity within cluster IV relative to others.

### Genetic diversity

The germplasm of fifty red rice populations collected by BRRI from several locations in Bangladesh were amplified using fifty SSR markers (Table 1). All of the markers produced 162 polymorphic bands, with an average of 3.24 and varied from 2 to 7 for each marker (Table 2). For the SSR loci, polymorphism information content (PIC) varied from 0.04 to 0.60 (with an average of 0.32) (Table 2). The observed genetic diversity index varied from 0.05 to 0.78 (with an average of 0.35). Major allele frequency varied from 30.77% to 98.00% and their size from 67 bp (RM413) to 285 bp (RM496). On average, 75.52% of the fifty red rice germplasm shared a common major allele at any locus. Those rare alleles, which have a frequency of less than 5%, were not identified at the fifty loci.

**Table 2:** Number of alleles, allele size range, allele frequency, unique allele, gene diversity and polymorphism information content (PIC) of 50 SSR markers across 50 red rice germplasms.

Marker	Chromosome		Motif	Allele(s)			Size Range (bp)	Size (bp)^b^	Allele Frequency (%)^c^	Gene Diversity^d^	PIC^e^
	#	Position (cM)		No.^a^	Unique	Rare					
**RM1**	1	29.7	(GA)26	4	1	0	87–112	112	57.14	0.55	0.47
**RM5**	1	94.9	GA)14	2	0	1	110–115	115	86.00	0.24	0.21
**RM35**	1	49.9	(GA)19	3	0	1	200–212	212	90.00	0.18	0.18
**RM312**	1	71.6	(ATTT)4(GT)9	2	0	0	98–104	98	90.00	0.18	0.16
**RM145**	2	49.8	-	2	0	0	165–172	165	94.00	0.11	0.11
**RM262**	2	103.3	(CT)16	4	0	1	127–152	152	82.00	0.32	0.30
**RM16**	3	131.5	(TCG)5(GA)16	3	2	0	170–202	170	96.00	0.08	0.08
**RM282**	3	100.6	(GA)15	2	1	0	126–135	135	98.00	0.05^min^	0.04^min^
**RM489**	3	29.2	(ATA)8	3	0	2	242–312	242	92.00	0.15	0.15
**RM554**	3	106.0	(GA)14	2	0	0	251–259	259	58.00	0.49	0.37
**RM518**	4	25.5	(TC)15	3	0	0	164–171	171	56.00	0.59	0.52
**RM537**	4	8.5	(CCG)9	2	0	1	215–221	221	96.00	0.08	0.07
**RM567**	4	153.6	(GA)21	2	0	0	280–289	280	90.00	0.18	0.16
**RM5473**	4	107.35	(TC)20	4	0	0	125–145	125	60.87	0.58	0.53
**RM39**	5	87.7	(CT)17CCA(TC)3	2	0	0	110–115	115	96.00	0.08	0.07
**RM178**	5	118.8	(GA)5(AG)8	2	0	0	117–123	117	81.63	0.30	0.25
**RM334**	5	141.8	(CTT)20	3	0	0	178–190	190	54.17	0.59	0.52
**RM413**	5	26.7	(AG)11	4	1	0	67–105	67^min^	79.59	0.35	0.33
**RM507**	5	-	(AAGA)7	2	0	0	244–253	253	86.00	0.24	0.21
**RM510**	6	20.8	(GA)15	2	0	0	213–223	223	94.00	0.11	0.11
**RM527**	6	61.2	(GA)17	2	0	0	212–220	220	78.00	0.34	0.28
**RM3330**	6	61.6	(CT)15	3	0	2	136–146	146	92.00	0.15	0.15
**RM70**	7	64.66	(ATT)33	4	0	0	185–211	185	58.00	0.59	0.54
**RM214**	7	34.7	(CT)14	4	0	0	116–144	116	50.00	0.66	0.60
**RM320**	7	62.5	(AT)11GTAT(GT)13	6	0	2	178–212	178	50.00	0.68	0.64
**RM542**	7	34.7	(CT)22	4	2	0	94–122	122	56.00	0.53	0.42
**RM478**	7	93.8	(AG)12	2	0	0	204–210	204	92.00	0.15	0.14
**RM500**	7	36.1	(AAG)9	2	0	0	251–257	257	60.00	0.48	0.36
**RM560**	7	54.2	(CT)12	2	1	0	130–150	150	98.00^max^	0.05^min^	0.04^min^
**RM342**	8	78.4	(CAT)12	3	1	0	132–152	152	82.00	0.30	0.27
**RM447**	8	124.6	(CTT)8	5	0	1	105–139	139	56.00	0.62	0.58
**RM215**	9	99.4	(CT)16	3	2	0	144–164	144	96.00	0.08	0.08
**RM105**	9	32.1	(CCT)6	3	1	0	133–143	133	86.00	0.25	0.22
**RM205**	9	114.7	(CT)25	4	1	0	111–146	111	68.00	0.49	0.44
**RM316**	9	1.8	(GT)8(TG)9(TTTG)4(TG)4	2	0	0	192–199	192	90.00	0.18	0.16
**RM464**	9	3.3	(AT)21	2	1	0	142–150	150	98.00	0.05	0.04
**RM285**	9	1.8	(GA)12	2	0	0	142–150	150	87.50	0.22	0.19
**RM304**	10	73.0	(GT)2(AT)10(GT)33	7	2	1	103–158	158	30.77^min^	0.78^max^	0.75^max^
**RM474**	10	-	(AT)13	4	0	0	215–248	103	60.00	0.58	0.53
**RM496**	10	113.0	(TC)14	4	0	0	246–285	285^max^	82.00	0.32	0.30
**RM591**	10	118.3	(AC)10	5	0	2	231–272	272	64.00	0.54	0.51
**RM21**	11	85.7	(GA)18	5	2	0	135–160	116	88.00	0.22	0.21
**RM206**	11	102.9	(CT)21	5	2	0	134–172	134	78.00	0.37	0.35
**RM224**	11	120.1	(AAG)8(AG)13	4	0	0	129–145	145	64.00	0.54	0.50
**RM287**	11	68.6	(GA)21	4	1	1	98–115	115	60.87	0.52	0.44
**RM536**	11	55.1	(CT)16	3	0	0	221–237	237	79.17	0.35	0.32
**RM20**	12	-	(ATT)14	3	0	0	152–163	152	60.00	0.55	0.48
**RM1337**	12	-	(AG)21	4	0	1	164–185	185	56.00	0.60	0.54
**RM5364**	12	-	TC)13	4	0	0	127–156	156	62.50	0.54	0.48
**RM7108**	12	-	(AGAT)8	4	1	2	169–191	169	56.00	0.54	0.45

^a^Total (162), Min (2), Max (7), Avg. (3.24)

^b^Avg. (167.96)

^c^Total (3776.21), Avg. (75.52)

^d^Total (17.67), Avg. (0.35)

^e^Total (15.87), Avg. (0.32)

Note: Major allele is described as the allele with the highest frequency. Rare alleles are described as alleles with a frequency less than 5%.

**Fig 5:**
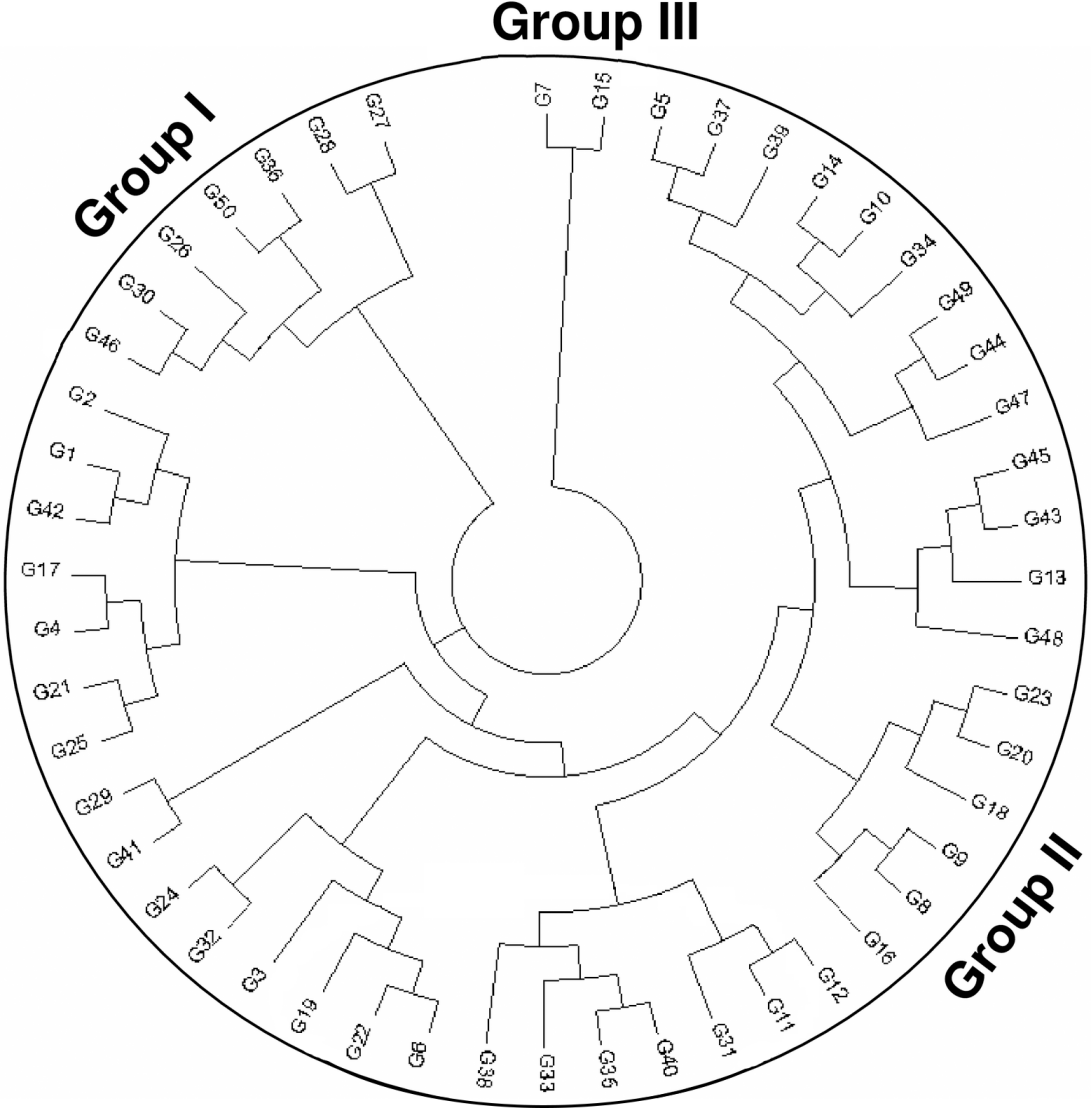
Dendogram of fifty red rice germplasm constructed from molecular data using MEGA software.

The study found 22 unique alleles detected in all tested germplasm (Table 2, see S1 Table for all of the unique alleles with their respective germplasm), and all fifty markers showed an allelic size-banding pattern, with a moderate observed genetic similarity coefficient of 0.59. Fig 5 gives the dendrogram generated through neighbor-joining (NJ) tree analysis, which grouped all germplasm into three major clusters I, II, and III, comprised of 7, 41, and 2 germplasms, respectively (see Table 1). Cluster II is further grouped into two sub-clusters, IIa and IIb, which themselves have further sub-groups.

### Model-based population structure

**Fig 6:**
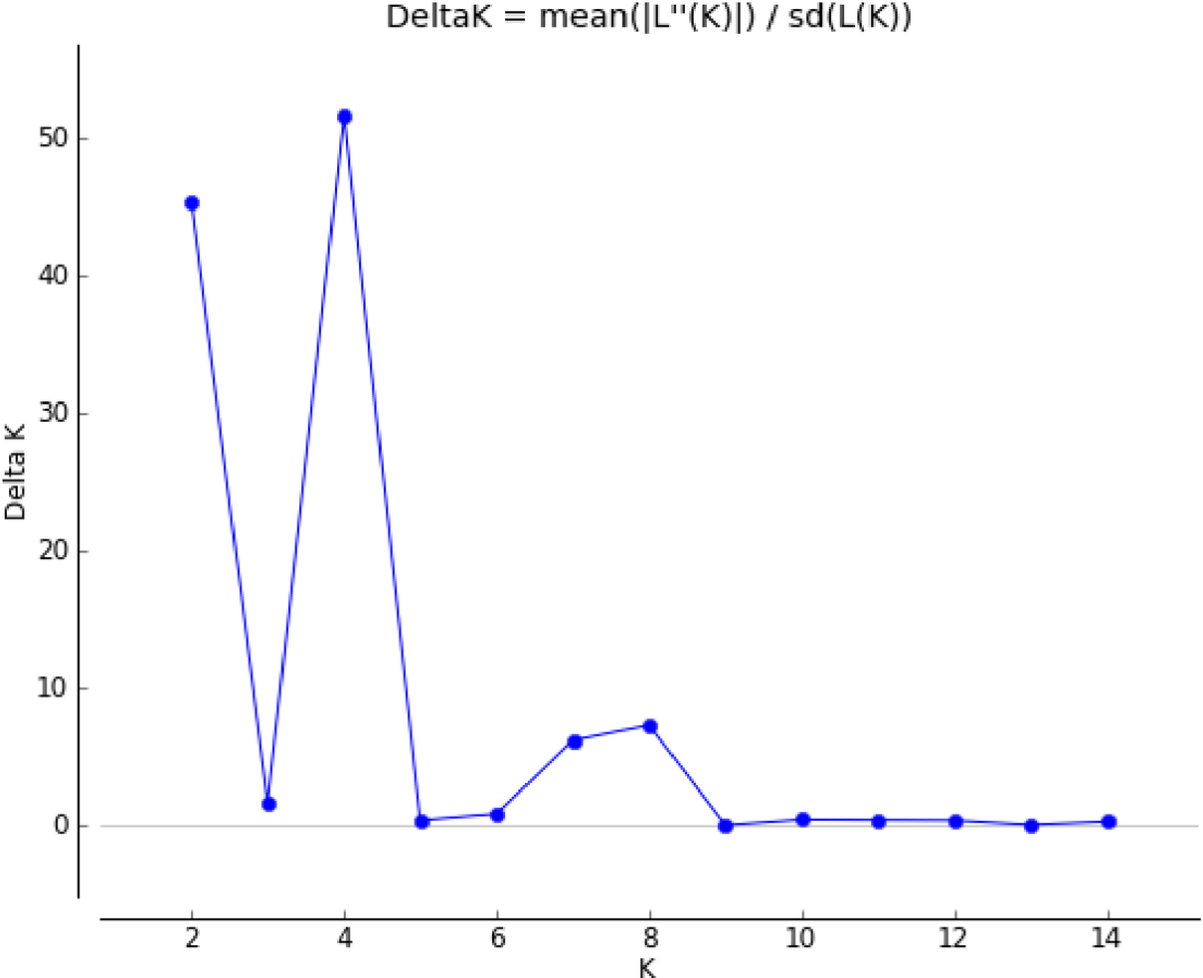
Estimation of population using LnP(D) derived ΔK for K from 1 to 15.

**Fig 7:**
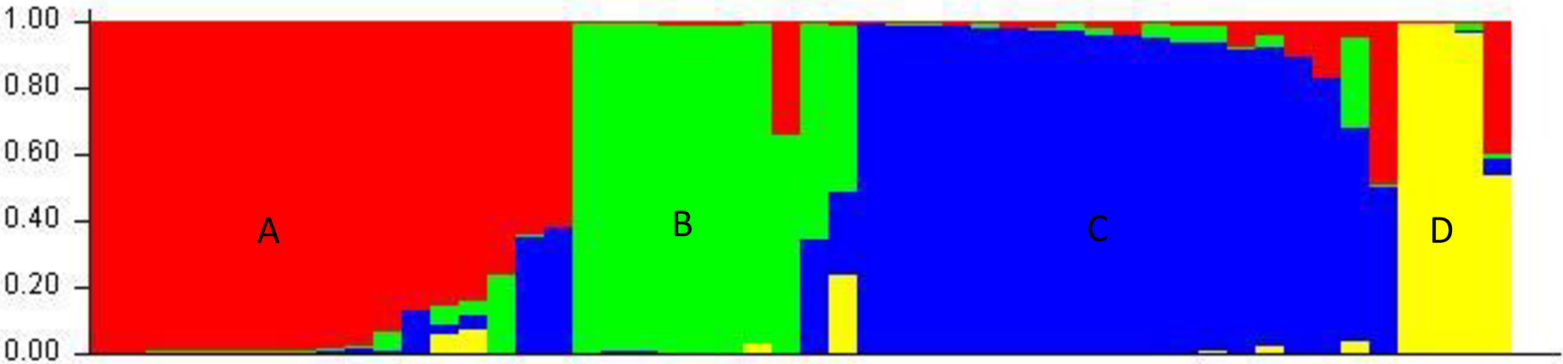
Assignment of fifty red rice germplasms into four populations (A, B, C and D) using STRUCTURE 2.3.4 software.

Bayesian analysis using STRUCTURE characterized the population structure for tested red rice germplasm. The LnP(D) as well Evanno’s ΔK values identified four genetically distinct populations (i.e., K = 4; Fig 6), A, B, C, and D (Fig 7), representing 34.60%, 19.40%, 37.90%, and 8.10% of germplasm, respectively. Membership fractions were used to classify these populations as pure or an admixture: population A showed ten pure (58.82%) and seven admixed (41.18%) individuals, B showed seven pure (70.00%) and three (30.00%) admixed individuals, C showed ten pure (52.63%) and nine (47.37%) admixed individuals, and D showed three pure (75.00%) and one (25.00%) admixed individuals.

Genetic variation by population was tested using FST statistics. The FST population values, by their degree of structuredness, were 0.4997 (A), 0.4226 (B), 0.3742 (D), and 0.3534 (C), with an average 0.4125, thus indicating a high population structure. We calculated specific FST values for populations A, B, C, and D, using STRUCTURE software. The average distances (i.e., expected heterozygosity) between the individuals in the same cluster were 0.2144 for A, 0.2831 for B, 0.3017 for C, and 0.4317 for D. The largest genetic (net nucleotide) distance (0.2240) was observed between A and D, and the lowest genetic distance was observed between A and C.

**Fig 8:**
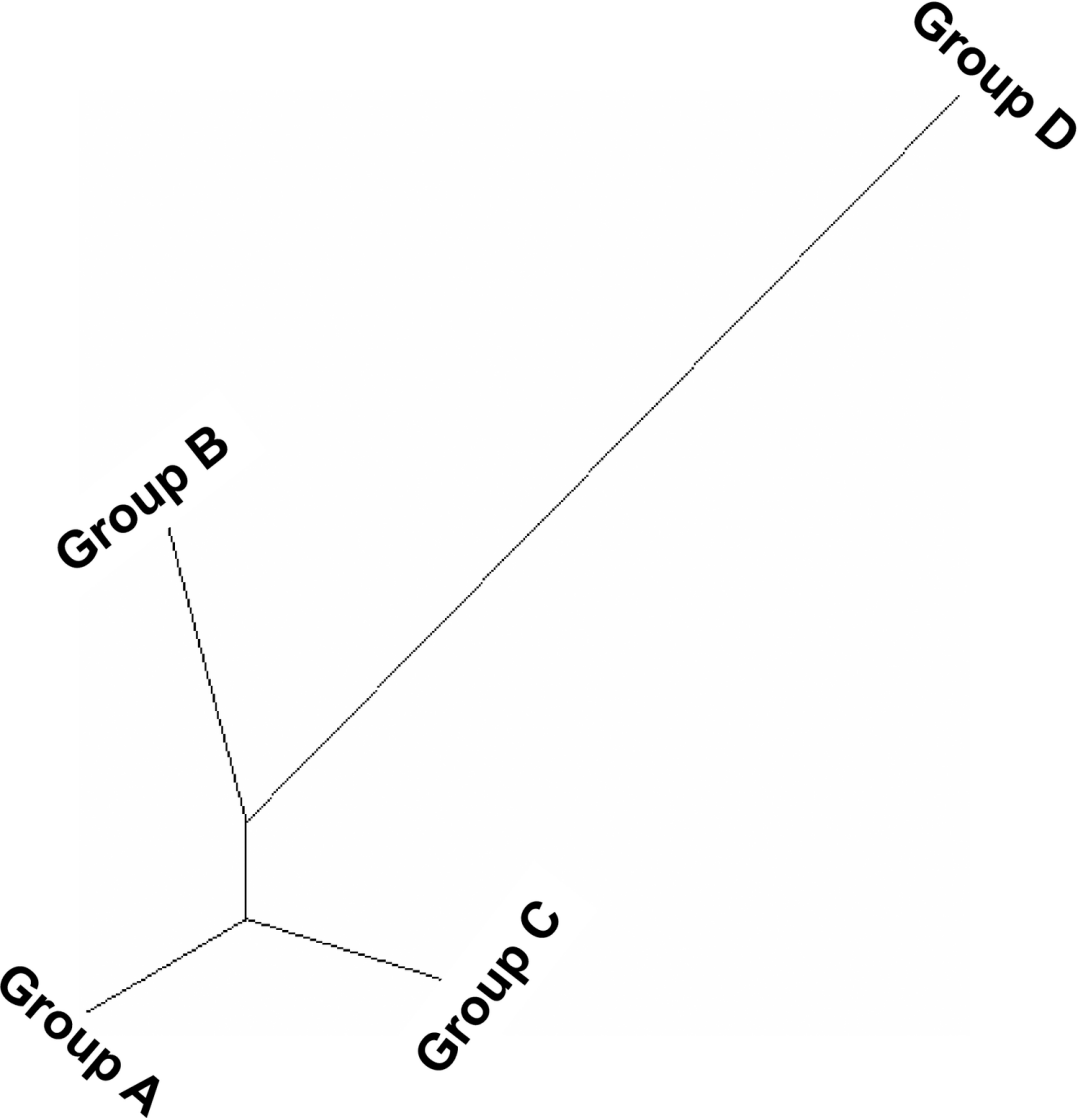
Neighbor-joining tree of fifty red rice germplasm based on populations obtained by the model-based approach.

**Fig 9:**
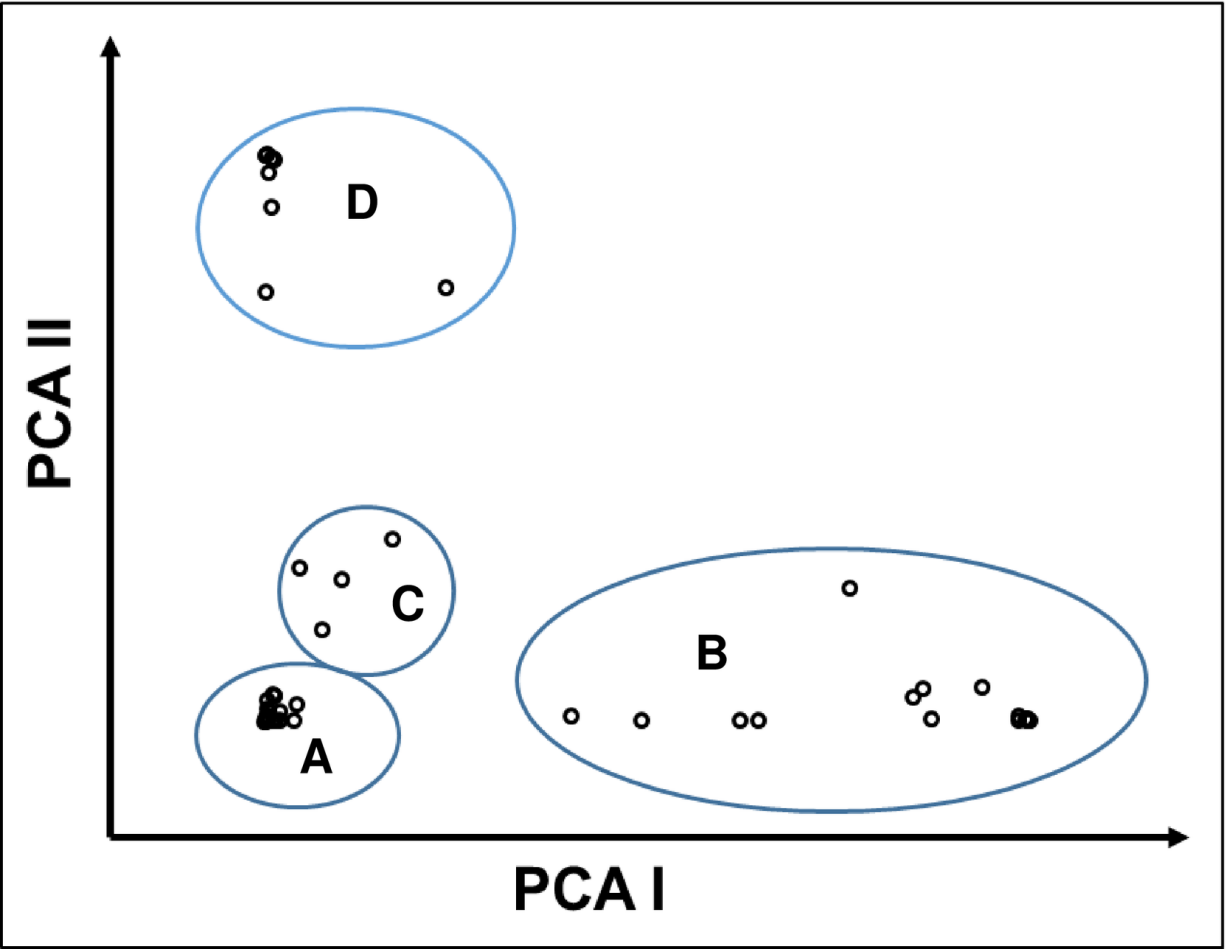
Principal component analysis (PCA) of fifty red rice germplasm based on population obtained by the model-based approach.

Both a neighbor-joining (NJ) tree and principal component analysis (PCA), based on population derived from the structure analysis, were also performed. Both NJ tree and PCA established the results obtained from structure analysis. The model-based population groups (A–D) were distinctly separated from each other (Fig 8). In the PCA analysis, the first two eigenvectors classified the germplasm in four distinct groups (Fig 9), which were similar to those observed in the structure analysis and NJ tree (Fig 8). Group I consisted of the higher yielding germplasm, suitable for future breeding programs, while group IV had lower yielding germplasm. Most of the population of group IV are dwarf and have a short growth phase.

### Analysis of molecular variance (AMOVA) from the model-based approach

**Fig 10:**
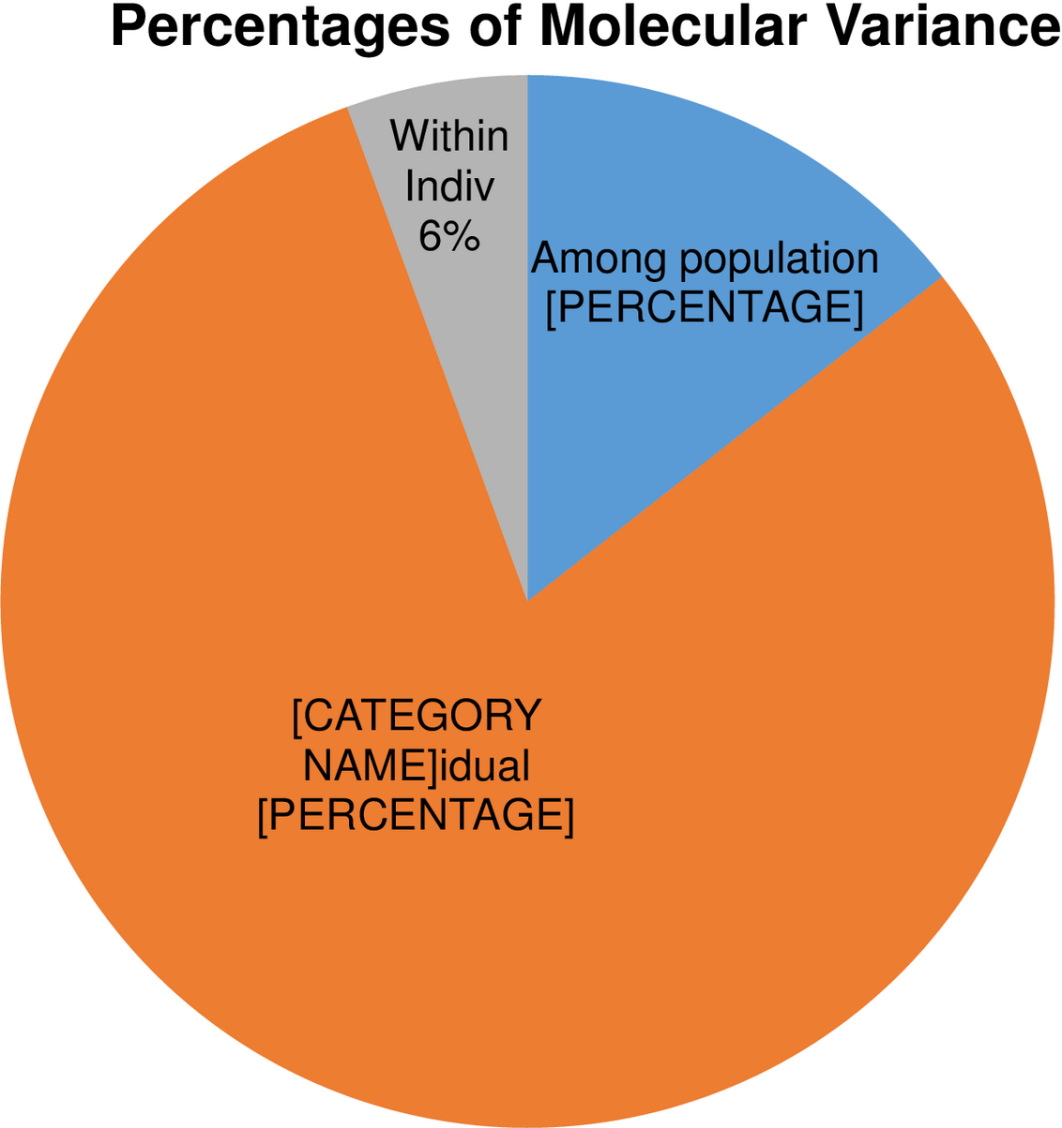
Analysis of molecular variance (AMOVA) of fifty red rice germplasm based on population obtained by the model-based approach.

The four populations obtained from structural analysis were subsequently exposed to AMOVA in order to determine the variation across and within populations. While a 14% variance was observed across populations, for individuals, 80% variance among and 6% within was observed (Fig 10 and Table 3). The pairwise FST values also demonstrated significant variation among populations, which varied from 0.108 to 0.207, suggesting that all of the groups were statistically distinguished from each other (Table 4). Populations A and D had the greatest difference from each other based on the FST test (Table 4).

**Table 3:** Analysis of molecular variance (AMOVA) of red rice available in Bangladesh.

Source	df	SS	MS	Est. Var.	% Variation
**Among population**	3	150.626	50.209	1.451	14%
**Among individual**	46	766.644	16.666	8.053	80%
**Within individual**	50	28.000	0.560	0.560	6%
**Total**	99	945.270		10.064	100%

**Notes**: df, degrees of freedom, SS, sum of squares, CV, variance component estimates, % total, percentage of total variation.

**Table 4:** FST Pair wise population differentiation according to groups of populations (using GenAlEx 6.503).

Population	A	B	C	D
**A**	0.000			
**B**	0.172**	0.000		
**C**	0.108**	0.138**	0.000	
**D**	0.207**	0.185**	0.179**	0.000

**Significance at P = 0.001 at 1,000 permutations

In general, the results from FST analysis and AMOVA both agreed with results obtained using the phylogenetic tree-based similarity coefficient distribution as well as the structure analysis, with all of these aforementioned approaches confirming the presence of both a statistically moderate genetic diversity and a high level of population structure. This represents a critical step for carrying out any future association mapping (AM) analysis.

## Discussion

In this study, we analyzed the diversity (both morphology and molecular) and population structure of fifty kinds of red rice that grow in different locations of Bangladesh in order to begin to lay a foundation for future yield-improvement rice breeding programs [58]. Based on agro-morphological data and fifteen quantitative traits, five groups (clusters I-V) were identified for the fifty germplasm. A majority of the most desirable characteristics were observed in cluster III, including higher yields (10.73 g/hill). Other good characteristics, from a breeding perspective, grouped in cluster IV (e.g., short growth duration, dwarf stature, and effective tiller number). This suggests that germplasm of cluster III and IV have the potential to be used for hybridization programs in order to generate higher yielding pre-breeding materials with a maximum probability of desirable traits.

Traits for culm diameter, plant height, panicle length, five panicles weight, and filled grains per panicle led the most to genetic diversity (S2 Table). The highest cluster means for leaf area index (cm^2^), culm diameter (mm), filled grains per panicle, and yield/hill (gm) were observed in cluster III. Cluster III also showed the lowest mean for 1000 grain weight (g). Cluster II showed the highest cluster means for the panicle length trait. The performance mean of the different clusters for the characteristics studied demonstrated that traits for dwarf stature, short growth duration, lower leaf area index (cm^2^), culm diameter (mm), panicle length (cm), filled grains per panicle, unfilled grains per panicle, and yield/hill (g) were clubbed in cluster IV along with the trait for maximum number of effective tiller.

Comparing these results to other research, the five clusters here, grouped using the Mahalanobis D^2^ statistic, resonates with Siddique et al. Siddique, Khalequzzaman, Islam, Ahamed and Rashid [59], who reported five and six clusters from forty traditional Boro and forty Aman rice germplasm also using D^2^ statistics. The clustering pattern in this study demonstrated that the germplasm constellated in the same cluster, albeit without originating from the same geographic area. Chakravorty, Ghosh and Sahu [60] evaluated fifty-one rice landraces and observed no specific relationship between geographical and genetic diversity.

Correlation analysis of genotypic and phenotypic traits can provide important information among study crop species or different groups of crop accessions [61]. Research has concentrated on genetic and morphological diversity analysis for both indigenous aromatic rice germplasm available in Bangladesh and the considerable phenotypic variation observed in a large number of aromatic rice germplasms [62]. In this study, the intra- (0.65–1.24) and inter- (3.70–16.31) cluster distances displayed a large range of diversity. This result resonates with Islam *et al*. [62], who measured ranges of intra- and inter-cluster distances from 0.61 to 1.27 and 3.71 to 16.12 in aromatic rice, respectively. Bhakal and Lal [63] suggest that having more distant clusters could afford hybridization in order to achieve a higher heterotic response. Results from the present study suggest that crosses between germplasm of cluster III and IV might provide high heterosis as well as transgressive segregation [64, 65].

Using SSR marker profiling to identify the genetic polymorphism available among the tested red rice germplasm, the measured average number of alleles per locus (3.24) compares with Islam [66], who studied 113 aromatic and fine rice germplasm from different districts of Bangladesh using forty-five SSR markers and detecting 3.11 alleles per locus. Further, Salgotra *et al*. [10] also detected 114 alleles with an average of 3.08 among 141 basmati accessions using SSR markers. In the present report, the PIC value ranged 0.04 to 0.75, with an average of 0.32. This accords with previous values: 0.34 to 0.88 and an average of 0.66 [46], as well as 0.04 to 0.59 [16] and 0.05 to 0.67 [66]. Ahmed, Khalequzzaman, Bashar and Shamsuddin [67], however, reported a range of 0.44 to 0.89 and average of 0.74, which is markedly higher than the results in the current study. While high PIC values can be attributed to the use of more informative markers [68], lower PIC values, in general, indicate that the germplasm under study are more closely related types. Higher values, by contrast, indicate greater diversity of materials. Results from this study suggest that these SSR markers may be useful tools for future genetic dissection of rice germplasm.

Identification of unique alleles can have great importance for breeding purposes [9, 69]. In this study, 22 unique alleles were identified by SSR markers (S1 Table). Each germplasm showed unique alleles for at least one microsatellite locus. However, unique alleles ranged from one to two at each locus [9]. Ten SSR markers amplified one unique allele and six markers amplified two unique alleles from 22 red rice germplasm (S1 Table). Moreover, germplasm with a higher number of unique alleles represents a potential bank of novel alleles for use in a crop improvement breeding program [15]. SSR markers have previously identified 58 unique alleles among rice, all found at 25 of 30 SSR loci [70]. Unique alleles have similarly been detected in both cultivated and wild rice [30, 71].

In the present study, genetic diversity ranged from 0.05 and 0.68, averaging 0.35 (Table 2), with a genetic similarity (GS) coefficient of 0.59, reflecting a moderate level of genetic diversity. This GS is comparable to Babu *et al*. [72], where an average GS of 0.55 was reported within 82 rice accessions. Jayamani, Negrao, Martins, Macas and Oliveira [73] reported a GS from 0.21 to 0.92 within 155 Japonica rice germplasms, and Yadav *et al*. [17] reported an average GS of 0.59 within 88 rice germplasm using fifty SSR markers. In Pakistan, a lower average GS of 0.39 was found for forty rice accessions [16].

Neighbor-joining tree analysis showed all fifty red rice germplasm separated into three major groups, with group II divided into further sub-groups. While the majority of the germplasm (82%) were in group II, and group III contained only the Surjamukhi and Noyantara germplasm, group II also had several sub-groups (Fig 4).

Population structure analysis displayed four populations A, B, C and D. The highest number of germplasm were grouped in population C, with only four—Honumanjata, Kharojamri, Khatomala and Laithajhora—in group D. Both AMOVA and FST analyses confirmed the population structure and its statistical significance (Tables 3 and 4). That the four groups were significantly different from each other was confirmed by pairwise FST, ranging from 0.108 to 0.207 (Table 4). Genetic variance among A–D of 14.00% and 80.00% was explained by individual distinctness within the population (Table 3). This finding is comparatively lower than other reports for variation within (64.72%) and among (35.28%) groups [74]. Zhang, Ni, Jiang, Deng, Qing, Yang *et al*. [75] similarly reported variation among (34%) and within (66%) populations.

Group I derived from STRUCTURE analysis showed higher yield than other groups, with group IV showing the lowest yield. Most of the population of group IV are short and have a lower growth phase that limits the yield and yield-contributing traits.

## Conclusion

In general, care should be taken when selecting germplasm for any breeding program attempting yield enhancement. Cluster analysis from the agro-morphological features grouped all the germplasm into five groups. Significant (at both p ≤ 0.05 and p ≤ 0.01) differences were found in all fifteen agro-morphological traits, which confirms the presence of diversification among the fifty red rice germplasm. From molecular analysis using SSR markers, three groups were found, while population structure analysis showed all tested germplasm constructed as four populations, each significantly differentiated and with their variation confirmed by AMOVA.

From the findings of the population structure analysis, in particular, large genetic differences were also observed between two populations (group A and D), suggesting that these could be crossed to improve grain yield. As such, the germplasm, as well as the highly polymorphic SSR markers identified in this study, have the potential to facilitate a red rice breeding program for enhanced yield. Based on these findings, we are currently developing such a program to facilitate more desirable, novel trait-linked markers usable for not only the mapping of QTLs/genes against a diversity of abiotic and biotic stresses but also the breeding of socially and economically desirable traits in red rice. The findings of this study provide a solid foundation for such further work in the effort to increase the yields of this increasingly popular and both medically and nutritively beneficial red rice.

## Supporting information

S1 Table Microsatellite loci that unique alleles in different test red rice germplasm. (DOC)

S2 Table Cluster mean and latent vectors for 15 quantitative characters of 50 red rice germplasm. (DOC)
