# Supplementary material for: Diversity and population structure of red rice germplasm in Bangladesh
Source: PLoS One. 2018 May 2;13(5):e0196096. doi: 10.1371/journal.pone.0196096 (PMC5931645; doi:10.1371/journal.pone.0196096)
Supplement: S1 Table — (DOC) [file pone.0196096.s001.doc]

**Supplementary information**

**S1 Table. Microsatellite loci that unique alleles in different test red rice germplasm**

| **Marker** | **Chro. No.** | **Unique allele (bp)** | **Name of germplasm** |
| --- | --- | --- | --- |
| RM1 | 01 | 93 bp | Matichak |
| RM16 | 03 | 170 bp and 202 bp | Laithajhora and Khatomala |
| RM282 | 03 | 126 bp | Laithajhora |
| RM413 | 05 | 67 bp | Binnatoha |
| RM542 | 07 | 115 bp and 122 bp | Binnatoha and Burikatari |
| RM560 | 07 | 253 bp | Khatomala |
| RM 342 | 08 | 132 bp | Kharojamri |
| RM215 | 09 | 144 bp and 164 bp | Kalisaita and Burikatari |
| RM105 | 09 | 143 bp | Shibjhota |
| RM205 | 09 | 111 bp | Kadamoni |
| RM464 | 09 | 161 bp | Dhalisaita |
| RM304 | 10 | 103 bp and 158 bp | Beursona and Khatomala |
| RM21 | 11 | 148 bp and 160bp | Khatomala and Kharojamri |
| RM206 | 11 | 149 bp and 178 bp | Burikatari and Boilam |
| RM287 | 11 | 110 bp | Khatomala |
| RM7108 | 12 | 169 bp | Honumanjata |
