# Supplementary material for: Diversity and population structure of red rice germplasm in Bangladesh
Source: PLoS One. 2018 May 2;13(5):e0196096. doi: 10.1371/journal.pone.0196096 (PMC5931645; doi:10.1371/journal.pone.0196096)
Supplement: S2 Table — (DOC) [file pone.0196096.s002.doc]

**Supplementary information**

**S2 Table. Cluster mean and latent vectors for 15 quantitative characters of 50 red rice germplasm**

|  | **I** | **II** | **III** | **IV** | **V** | **Vector 1** | **Vector 2** |
| --- | --- | --- | --- | --- | --- | --- | --- |
| Leaf area index (cm^2^) | 49.43 | 63.43 | 78.43 | 38.48 | 45.53 | 0.3881 | 0.1836 |
| Culm diameter (mm) | 3.96 | 4.49 | 4.65 | 3.48 | 3.94 | 0.3362 | 0.1973 |
| Effective tiller number | 7.20 | 7.00 | 7.33 | 7.75 | 7.05 | -0.1891 | 0.0003 |
| Plant height (cm) | 118.04 | 120.40 | 120.30 | 110.48 | 116.81 | 0.1879 | 0.2368 |
| Days to flowering | 79.85 | 77.37 | 75.83 | 68.47 | 70.08 | 0.2357 | -0.3234 |
| Days to maturity | 107.60 | 104.56 | 104.00 | 96.28 | 97.28 | 0.2261 | -0.3467 |
| Panicle length (cm) | 22.96 | 23.78 | 23.30 | 21.16 | 23.42 | 0.2604 | 0.1971 |
| Five panicles weight (g) | 7.57 | 11.05 | 12.23 | 8.59 | 13.37 | 0.0702 | 0.2164 |
| Filled grains per panicle | 73.90 | 120.62 | 176.67 | 65.63 | 90.38 | 0.4037 | 0.0924 |
| Unfilled grains per panicle | 21.40 | 19.00 | 21.00 | 15.81 | 17.62 | 0.1950 | -0.1098 |
| Grain length (mm) | 8.56 | 8.15 | 7.55 | 8.44 | 8.44 | -0.2053 | -0.2009 |
| Grain breadth (mm) | 2.92 | 2.83 | 2.73 | 3.01 | 3.05 | -0.2042 | 0.4663 |
| Length-breath ratio | 2.96 | 2.92 | 2.77 | 2.82 | 2.80 | 0.0640 | -0.4938 |
| 1000 grain weight (g) | 26.54 | 21.97 | 20.20 | 26.03 | 25.69 | -0.3196 | 0.1961 |
| Yield/hill (g) | 7.89 | 9.16 | 10.73 | 6.47 | 7.81 | -0.3030 | 0.0144 |
